# Antibacterial Activity of a Phenylpropanoid from the Root Extract of *Carduus leptacanthus* Fresen

**DOI:** 10.1155/2023/4983608

**Published:** 2023-09-06

**Authors:** Abebe Dagne, Sileshi Degu, Abiy Abebe, Daniel Bisrat

**Affiliations:** ^1^Department of Pharmacy, College of Health Sciences, Debre Markos University, Debre Markos, Ethiopia; ^2^Traditional and Modern Medicine Research Directorate, Ethiopian Public Health Institute, Addis Ababa, Ethiopia; ^3^Department of Pharmaceutical Chemistry and Pharmacognosy, School of Pharmacy, College of Health Sciences, Addis Ababa University, Addis Ababa, Ethiopia

## Abstract

**Background:**

The emergence and rapid spread of antimicrobial drug-resistance microorganisms exacerbate the treatment of infectious diseases, underscoring the importance of finding new, safe, and effective drugs. In Ethiopia, the roots of *Carduus leptacanthus* have traditionally been employed to treat microbial infectious diseases The aim of this study was to evaluate the antibacterial activity of the root extract and its primary components against six bacterial strains (*Staphylococcus aureus*, *Staphylococcus epidermidis*, *Streptococcus agalactiae*, *Escherichia coli*, *Pseudomonas aeruginosa*, and *Klebsiella pneumonia*).

**Methods:**

The extraction involved maceration of air-dried and powdered roots of *C. leptacanthus* with 80% methanol. The compound was isolated from the root extract using silica gel column chromatography and recrystallization in CHCl_3_/MeOH (9 : 1) and was characterized using ESI-MS and 1D-NMR spectroscopy. Antibacterial activity of the extract was assessed using agar well diffusion and broth microdilution methods.

**Results:**

Syringin, a phenylpropanoid, was isolated and characterized from the extract of *C. leptacanthus*. The extract showed the most substantial efficacy against *S. epidermidis* (MIC = 5.33 mg/ml and inhibition zone diameter of 24 mm at 200 mg/m). Syringin also elicited antibacterial activity against *S. aureus* (MIC = 13.33 mg/ml), *S. epidermidis* (MIC = 16 mg/ml), and *S. agalactiae* (MIC = 16 mg/ml). Despite being tested up to a maximum concentration of 16 mg/ml, syringin did not exhibit antibacterial activity against the Gram-negative bacteria (*P. aeruginosa*, *E. coli*, and *K. pneumonia*).

**Conclusions:**

In conclusion, the findings suggest that syringin exhibits partial involvement in the root extract's antibacterial activity, thereby potentially supporting the traditional medicinal use of the plant.

## 1. Background

Infectious diseases are the main threats that lead to morbidity and mortality in the world, particularly in the developing countries [1]. Antimicrobial drug resistance and side effects have become major sources of morbidity and mortality worldwide. Thus, searching for new, effective, and safe antimicrobial agents to mitigate the wide spread of drug resistance has become indispensable. Thus, natural products, including secondary metabolites from medicinal plants, are sources of a new drug. Utilization of medicinal plants for the variety of ailments by the human beings was the oldest one as they contain different class of compounds, such as tannins, alkaloids, carbohydrates, terpenoids, steroids, and flavonoids [2, 3].

The genus *Carduus* (family Asteraceae) comprises about 90 species [4, 5] that occurs mainly in Europe, Central Asia, West Asia, Eurasia, Africa, Northern Africa, and the Mediterranean region [5–7]. *Carduus leptacanthus* is locally known as *Guccino* (in Afan Oromo), and its root parts are traditionally used for the treatment of infectious diseases and pain in Ethiopia. It was also reported that the powdered dry stem of *C. leptacanthus* is used for the treatment of ascariasis and hemorrhoids [2, 8–10]. However, the roots of *C. leptacanthus* have not been previously subjected to any chemical and biological investigations. The present study has been conducted to evaluate the antibacterial activity of 80% methanol root extract of *C. leptacanthus* and its major constituents.

## 2. Materials and Methods

### 2.1. Chemicals and Reagents

Distilled water (Pharmaceutics laboratory, AAU), methanol (CarloErba, France), and chloroform (Sigma-Aldrich Co., MO, USA) were employed for extraction and isolation. TLC was accomplished on a precoated aluminium backed silica gel 60 F_254_ plates, with 0.20 mm layer thickness (Merck KGaA, Darmstadt, Germany). Column silica gel (60 F_254_, 70–240 mesh, Merck KGaA, Darmstadt, Germany) was used for chromatographic separation. In addition to this, ciprofloxacin disk, MHA (Oxoid Ltd, Basingstoke, Hampshire, England), MHB (Oxoid Ltd, Basingstoke, Hampshire, England), gloves, and 2,3,5-triphenyltetrazolium chloride were used. The chemicals and reagents that were used in this study were of laboratory grade.

### 2.2. Instruments

Organic solvents were removed using a rotary evaporator R-200 (Buchi, Switzerland). UV cabinet (CAMAG, Muttenz, Switzerland) was used to view the TLC chromatograms. ^1^H-NMR and ^13^C-NMR spectra were recorded at room temperature on a Bruker Avance DMX400 FT-NMR spectrometer (Bruker, Billerica, MA, USA), operating at 400 MHz for ^1^H and 100 MHz for ^13^C at room temperature using deuterated MeOD-d_4_, with tetramethylsilane (TMS) as the internal standard. Spin multiplicities were reported as following: *s* (singlet), *d* (doublet), *dd* (doublet of doublets), and *dt* (doublet of triplets). ESI-MS were recorded on an Ultimate 3000LC-MS. The measurement was carried out by an electrospray ionization method in a positive mode with the source voltage and temperature being fixed at 3 kV and 250°C.

### 2.3. Plant Materials

The roots of *C. leptacanthus* Fresen were collected from Machakel Woreda, East Gojjam Zone, Amhara Regional State, around 327 km North of Addis Ababa, Ethiopia. Authentication of the plant was conducted by Mr. Melaku Wondaferash, at the Department of Biology, National Herbarium, Ethiopia, where the specimens were deposited giving a voucher number (AD004).

### 2.4. Extraction

The roots were cleaned of dust and debris, washed gently with water, and air-dried under shade for two weeks. Then, the dried roots were pulverized with a grinder to reduce to an appropriate size. Dried and powdered roots (200 g) were macerated in 80% methanol at room temperature with occasional shaking for 72 h. It was then first filtered using nylon cloth, followed by Whatman filter paper no. 1. The residue was remacerated for another 72 h twice. Then, the combined filtrates were evaporated under reduced pressure using a rotary evaporator. The extract was further concentrated to dryness with oven at 40°C. Then, the root extract was weighed (24.8 g) and stored in a refrigerator in air tight plastic containers until used.

### 2.5. Compound Isolation

Column chromatography was employed for compound isolation. The column was initially packed with slurry, which was prepared by mixing silica gel (75 g) in chloroform (100 ml). The root extract (3.3 g) was adsorbed on silica gel by adding root extract and silica gel (2.5 g) in methanol. The mixture was then concentrated with a rotary evaporator until dried. The adsorbed sample was then loaded on the top of the column and eluted with gradual increase of methanol in chloroform. A total of 162 fractions were collected, of which fractions 121–162 eluted with CHCl_3_/MeOH (9 : 1; 400 ml) resulted in a white-plate crystal (30 mg, coded as CL-1). The purity of the compound was monitored by TLC when viewed under ultraviolet (UV) light of wavelengths 254 and 366 nm. For the TLC analysis, the process began by taking an individual sample weighting 1 mg and dissolved it in 0.5 ml of methanol. The solution (10 *μ*l) was then carefully spotted onto TLC plate using a TLC applicator. The plate was then transferred to a TLC chamber, which contained a mixture of CHCl_3_/MeOH in a ratio of 9 : 1 as a mobile solvent system.

### 2.6. Bacterial Strains

Six bacteria including *E. coli* (ATCC 25922), *P. aeruginosa* (ATCC 27853), *S. aureus* (ATCC, 25923), *S. epidermidis* (ATCC 12228), *Streptococcus agalactiae* (ATCC 12386), and *K. pneumoniae* (ATCC 700603) were obtained from the Ethiopian Health and Nutrition Research Institute particularly from the microbiology laboratory of Traditional and Modern Medicine Drug Research Directorate (TMMRD).

### 2.7. Evaluation of Antibacterial Activity

#### 2.7.1. Media and Inoculum Preparation

MHA was used to subculture the bacteria [11]. Well-isolated colonies (3–5) of the same morphological type from an agar plate culture were selected [12]. The colonies of bacteria were transferred to the broth using loop. For this, a UV spectrophotometer was used to adjust the bacterial suspension by measuring its absorbance with 1 cm path length at the wavelength of 625 nm. So, the absorbance should be in the range from 0.08 to 0.10 which is proportional to 1 × 10^8^ CFU/ml bacteria [13, 14]. This inoculum suspension was diluted in 1 : 10 to get 1 × 10^7^ CFU/ml to determine the agar well diffusion assay [12].

#### 2.7.2. Agar Well Diffusion Method

A volume of the microbial inoculum was spread over the entire agar surface. After solidifying, a sterile cork borer aseptically punched the streaked plate to form holes with the diameter of 8 mm. 100 *µ*L of the standard drug, the negative control, and extract solution at desired concentrations were introduced into the wells. The plates were incubated at 37°C for 18–24 h, at 28°C for 48 h. The zones of inhibition diameters in mm were measured using a ruler, and the average values were calculated [15, 16]. The antibacterial activity of the extract was evaluated by comparing its inhibition diameter with the standards [17]. Ciprofloxacin 5 *µ*g was used as a positive control, while 1% DMSO was used as a negative control. Each assay was carried out in triplicate.

#### 2.7.3. Determination of Minimum Inhibitory Concentration

The minimum inhibitory concentration (MIC) of the crude extracts and the isolated compound were evaluated by the broth microdilution method using 96-well plates according to the Clinical and Laboratory Standards Institute [18]. A stock solution of the respective plant extract (128 mg/ml) and the isolated compound (32 mg/ml) were prepared by dissolving in 1% DMSO. Two-fold serial dilutions with multichannel micropipettes were made down the column from 64 mg/ml to 0.05 mg/ml (extract) and 16 mg/ml to 0.05 mg/ml (compound). The bacterial suspension containing approximately 5 × 10^5^ CFU/ml was prepared from a refreshed culture. From this suspension, 100 *μ*l was inoculated into each well and incubated. Sterility control was put on the last column. After incubation, 40 *μ*l of a 0.2 mg/ml solution of 2,3,5-triphenyltetrazolium chloride was added to each well as an indicator of microbial growth and incubated at 37°C for 30 min. After incubation, the MIC values were visually determined by observing the presence or absence of pink color. The lowest concentration of each extract displaying no visible pink color was recorded as the MIC. MIC values were determined in triplicate [19, 20].

### 2.8. Data Analysis

The data were analyzed using SPSS (Statistical package for social science) software version 25.0. The results were presented as the mean ± standard error of the mean. One-way analysis of variance (ANOVA) followed by Tukey's post hoc test was used to compare differences in mean among the treatment and control groups. *p* values <0.01 were considered statistically significant.

## 3. Results and Discussion

### 3.1. Structural Elucidation of the Isolated Compound

The root extract of *C. leptacanthus* underwent a phytochemical investigation over silica gel column chromatography, which was followed by recrystallization in CHCl_3_/MeOH (9 : 1), resulting in the isolation of a white-plate crystal (**1**). Compound **1** displayed a retention factor (*R*_*f*_) value of 0.6 in CHCl_3_/CH_3_OH (4 : 1) solvent system. The positive-mode electrospray ionization mass spectrum (ESI-MS) of compound **1** showed a pseudomolecular ion at m/z = 395.1321 [M + Na]^+^, corresponding to a molecular formula of C_17_H_24_O_9_ (calcd. m/z = 395.1318 [M + Na]^+^).

Analysis of the ^1^H-NMR spectrum indicated the presence of a phenylpropanoid skeleton, with proton signals resonating at *δ*6.32 (1H, *dt*, *J*_1_ = 8 Hz, *J*_2_ = 18 Hz, H-8), *δ*6.55 (1H, *d*, *J* = 16 Hz, H-7), and *δ*4.22 (2H, *dd*, *J* = 8 Hz, H-9), as well as a singlet signal detected at 6.75 ppm due to two equivalent aromatic methines, corresponding to H-3 and H-5 protons. The ^1^H-NMR spectrum also revealed two equivalent methoxy groups, observed as a singlet signal resonating at 3.85 ppm and integrated for six protons. An anomeric proton at *δ*4.86 (1H, overlap, H-1′) and six additional signals resonating between 3.40 and 3.91 ppm the ^1^H-NMR spectrum suggested the presence of an *O*-glucose moiety in the compound.

**Figure 1:**
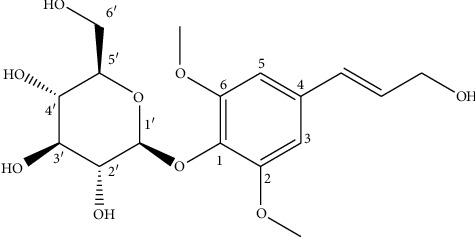
The chemical structure of syringin isolated from *Carduus leptacanthus*.

The ^13^C-NMR spectrum of compound **1** displayed the presence of 17 carbon atoms, with 13 carbon signals being observed in the DEPT-135 spectrum, including two equivalent aromatic methines, two trans-coupling olefinic methines, oxymethylene, and two methoxy groups. The presence of the O-glucose moiety was also confirmed by the observation of signals at *δ*105.31, C-1′; 62.57, C-6′; 71.32, C-4′; 75.71, C-2′; 77.82, C-5′; 78.35, C-3′ in the ^13^C-NMR spectrum of compound **1**. Analysis of the spectral data of compound **1** (see below for complete assignments of both ^1^H and ^13^C signals) and comparison to previously reported data led to the identification of compound **1** as syringin (Figure 1), in agreement Yang [21].

CL-1: white-plate crystal (yield = 0.91% from the root extract); *R*_*f*_ value of 0.6 (CHCl_3_/CH_3_OH; 4 : 1); +ve ESI-MS (Figure S1): m/z = 395.1321 [M + Na]^+^, corresponding to a molecular formula of C_17_H_24_O_9_ (calcd. m/z = 395.1318 [M + Na]^+^); ^1^H-NMR (Figure S2): 3.40 (H-4′, *dd*, 1H); 3.49 (H-3′, *dd*, 1H); 3.54 (H-6′, *d*, 2H); 3.76 (H-5′, *dt*, 1H); 3.85 (2 –OCH_3_; 6H, *s*); 3.91 (H-2′, *dd*, 1H); 4.22 (H-9, 2H, *dd*, *J* = 8 Hz); 4.86 (H-1″, 1H, overlap); 6.32 (H-8, 1H, *dt*, *J* = 16, 8 Hz); 6.55 (H-7, 1H, *d*, *J* = 16 Hz); 6.75 (H-3 & H-5, 2H, *s*). ^13^C-NMR (Figure S3) & DEPT-135 (Figure S4): 57.00 (2 × OCH_3_); 62.57 (C-6′); 63.56 (C-9); 71.32 (C-4′); 75.71 (C-2′); 77.82 (C-5′); 78.35 (C-3′); 105.31 (C-1′); 105.43 (C-3 & C-5); 130.01 (C-8); 131.25 (C-7); 154.33 (C-2 & C-6); 135.24 (C-4); 135.85 (C-1). ^1^H and ^13^C-NMR spectral data of CL-1 were in a good agreement with syringin [21].

### 3.2. Antibacterial Activity of the Extract

In Ethiopia, the root parts of *C. leptacanthus* have been traditionally used to treat infectious diseases. To assess its traditional medicinal claim, the root extract was tested against six bacterial strains using the agar well diffusion method to determine the inhibition zone diameter (Table 1). The root extract demonstrated antibacterial activity against most of the tested bacterial strains at a concentration of 100 mg/ml (Table 1), with *S. aureus* (zone diameter inhibition of 16.67 ± 0.33 mm) being the most susceptible strain, followed by *S. epidermidis* (with a zone diameter inhibition of 16.00 ± 0.58 mm). *S. aureus* can lead to various serious ailments of the human skin and even life-threatening infections, while *S. epidermidis* is a frequently occurring species that can cause infective endocarditis [22]. However, the root extract did not exhibit antibacterial activity against *S. agalactiae* at 100 mg/ml, but increased concentration up to 200 mg/ml resulted in improved activity.

The root extract showed inhibitory activity against *S. epidermidis* and *S. agalactiae* at 200 mg/ml, with the mean inhibition zone diameter of 24.00 ± 0.58 and 10.67 ± 0.33 mm, respectively. A previous study conducted by Muhaisen [23] on *C. marianium* L. also showed that the methanolic extract produced a clear inhibition zone diameter of 13–18 mm against *S. aureus*, but the aqueous extract was ineffective against *S. aureus*. The root extract at 100 and 200 mg/ml did not have any effect on Gram-negative bacteria (*P. aeruginosa*, *E. coli*, and *K. pneumonia*) as it was evident by an equal level of zone inhibition with the negative control. However, in other studies, the root extract of *C. macracanthus* suppressed the growth *E. coli* at a concentration of 50 mg/mL [24]. Notably, different studies have reported varying effects of *C. leptacanthus* extracts on bacteria, which may be attributed to differences in their constituents. *E. coli* has been reported as one of the predominant pathogens associated with hospital-acquired neonatal ocular infections, as well as causing various diseases ranging from less severe diarrhea to a grave condition termed hemolytic uremic syndrome [25].

According to the data presented in Table 1, the MIC values of the root extract against the six tested bacterial strains ranged from 5.33 to 32 mg/ml. Of these strains, *S. epidermidis* was the most susceptible to the root extract with an MIC value of 5.33 mg/ml, while *E. coli* demonstrated the least susceptible with an MIC value of 32 mg/ml. Strong susceptibility (*p* < 0.01) to the root extract was observed in *S. epidermidis* (MIC = 5.33 ± 1.33 mg/ml) and *S. agalactiae* (MIC = 6.67 ± 1.33 mg/ml) compared to the isolated compound, syringin. This disparity may be due to the presence of some other additional compounds in the root extract or synergetic effect between constituents. MIC values are used to determine the efficacy of an extract against a particular bacterial species, and the cut-off value varies depending on the strain tested. There is no universally accepted cut-off value for antibacterial activity of plant extracts. The MIC value of ≤8000 *μ*g/mL is generally considered as an indicator of antibacterial activity, while a value of ≥8000 *μ*g/mL indicates weak or no activity [26]. Thus, in this study, the root extract exhibited antibacterial activity against *S. epidermidis*, *S. agalactiae*, and *S. aureus*, while syringin displayed weak antibacterial activity against these same strains. Nevertheless, there was no notable difference observed between the root extract and syringin when tested against *E. coli*, *P. aeruginosa*, and *K. pneumonia.* These pathogenic strains are commonly associated with nosocomial infections, such as bloodstream infections, chronic obstructive pulmonary disease (COPD), cystic fibrosis, and ventilator-associated pneumonia (VAP) [25, 27].

For the first time, this study identified syringin, a phenylpropanoid glycoside, in the root of *C. leptacanthus*. Syringin demonstrated antibacterial activity against *S. aureus* (MIC = 13.33 mg/ml), *S. epidermidis* (MIC = 16 mg/ml), and *S. agalactiae* (MIC = 16 mg/ml). Pan et al. [28] suggested that syringin's phenolic di-methoxy group may contribute to the antibacterial activity of the compound. Furthermore, the inhibition of antibiotic efflux pump and bacterial cell wall synthesis could be the mechanism by syringin exerts its antibacterial activity [29]. However, when tested against Gram-negative bacteria (*P. aeruginosa*, *E. coli*, and *K. pneumonia*), syringin did not demonstrate any antibacterial activity. Generally, Gram-positive bacteria are more sensitive to antimicrobial substances due to the structural component of their cell wall [30]. As such, both the root extract and syringin were more effective against Gram-positive strains than Gram-negative strains.

Syringin has been isolated from other *Carduus* species, including *C. schimperi* [31] and *C. chevallieri* [32]. The *Carduus* genus is known for its rich source of bioactive secondary metabolites of great importance. Up to now, more than 80 secondary metabolites belonging to diverse structural types of lignans, flavonoids, coumarins, alkaloids, sterols, triterpenes, volatile oils, and polyphenols have been identified [33–38]. Syringin has also been detected in other plants, including *Fraxinus rhynchophylla* [21], *Musa paradisiaca* [39], *Centaurea bella* Trautv, and many other species [40]. Syringin isolated from *Stevia rebaudiana* (Bert.) demonstrated efficacy against various bacterial species, including *B. cereus*, *B. megaterium*, *B. aureus*, *Sarcina lutea*, *E. coli*, *Salmonella paratyphi*, *Salmonella typhi*, *Shigella dysenteriae*, and *Vibrio parahaemolyticus.* However, it was ineffective against *B. subtilis*, *P. aeruginosa, Shigella boydii*, and *Vibrio mimicus* [33]. In another study, syringin from *Fagus sylvatica* L showed antibacterial activity [41].

## 4. Conclusions

The current research involved an examination of the phytochemicals present in the root extract of *C. leptacanthus*, resulting in the identification of syringin for the first time. The root extract possessed greater effectiveness against the Gram-positive bacterial strains than the Gram-negative bacterial strains. Within the Gram-positive strains, the root extract demonstrated particularly strong antibacterial activity against *S. aureus*, followed by *S. epidermidis*. While syringin elicited a moderate antibacterial activity against *S. aureus*, *S. epidermidis*, and *S. agalactiae*, it was less potent than the root extract. Nevertheless, these findings provide evidence that the presence of syringin partially accounts for the antibacterial effects of the root extract. We suggest that future studies should attempt to isolate additional compounds given the comparatively lower activity of syringin. Overall, this study advances the traditional claims regarding the plant's ability to infectious diseases.

## Figures and Tables

**Table 1:** Zone of inhibition and minimum inhibitory concentrations of root extract and compound isolated from *Carduus leptacanthus*.

Bacterial strains	Diameter of zone of inhibition in mm, DZI (mean ± SEM)				MIC (mg/ml)	
	Root extract (100 mg/ml)	Root extract (200 mg/ml)	Ciprofloxacin (5 *µ*g)	1% DMSO	Root extract	Syringin
*S. aureus*	16.67 ± 0.33^a^	18 ± 0.58^b^	23.67 ± 0.33^c^	8.00 ± 0.0^d^	8.00 ± 0.0^d^	13.33 ± 2.67^e^
*S. epidermidis*	16.00 ± 0.58^a^	24 ± 0.58^b^	33.00 ± 0.0^c^	8.00 ± 0.0^d^	5.33 ± 1.33^d^	16.00 ± 0.0^e^
*S. agalactiae*	9.00 ± 0.0^a^	10.67 ± 0.33^a^	21.67 ± 0.33^b^	8.00 ± 0.0^a^	6.67 ± 1.33^d^	16.00 ± 0.0^e^
*E. coli*	8.00 ± 0.0^a^	8.00 ± 0.0^a^	32.67 ± 0.33^b^	8.00 ± 0.0^a^	32.00 ± 0.0	NA
*P. aeruginosa*	8.00 ± 0.0^a^	8.00 ± 0.0^a^	32.67 ± 0.33^b^	8.00 ± 0.0^a^	16.00 ± 0.0	NA
*K. pneumonia*	8.00 ± 0.0^a^	8.00 ± 0.0^a^	23.00 ± 0.0^b^	8.00 ± 0.0^a^	13.33 ± 2.67	NA

*Note*. Values are presented as the mean ± SEM; *n* = 3; means followed by a different letter indicate significant differences between different doses of the same treatments and negative control and positive control in the same row (*p* < 0.01) for DZI and MIC separately; NA = not active up to the concentration of 16 mg/ml.

## Data Availability

The data used to support the findings of this study are available from the corresponding author upon request.

## Abbreviations

CFU: Colony forming unit
DEPT: Distortional enhancement by polarization transfer
DMSO: Dimethyl sulfoxide
MHA: Muller Hinton agar
MHB: Muller Hinton broth
MIC: Minimum inhibitory concentration
SEM: Standard error of the mean
TLC: Thin layer chromatography.

## Supplementary Materials

Supplementary Materials Figure S1: positive-mode electrospray ionization mass spectrum (ESI-MS (+-mode)) of CL-1. Figure S2: ^1^H-NMR spectrum of CL-1. Figure S3: ^13^C-NMR spectrum of CL-1. Figure S4: DEPT-135 spectrum of CL-1.
