# Supplementary material for: Antibacterial Activity of a Phenylpropanoid from the Root Extract of Carduus leptacanthus Fresen
Source: J Trop Med. 2023 Sep 6;2023:4983608. doi: 10.1155/2023/4983608 (PMC10499531; doi:10.1155/2023/4983608)
Supplement: Supplementary Materials — Figure S1: positive-mode electrospray ionization mass spectrum (ESI-MS (+-mode)) of CL-1. Figure S2: 1H-NMR spectrum of CL-1. Figure S3: 13C-NMR spectrum of CL-1. Figure S4: DEPT-135 spectrum of CL-1. [file 4983608.f1.docx]

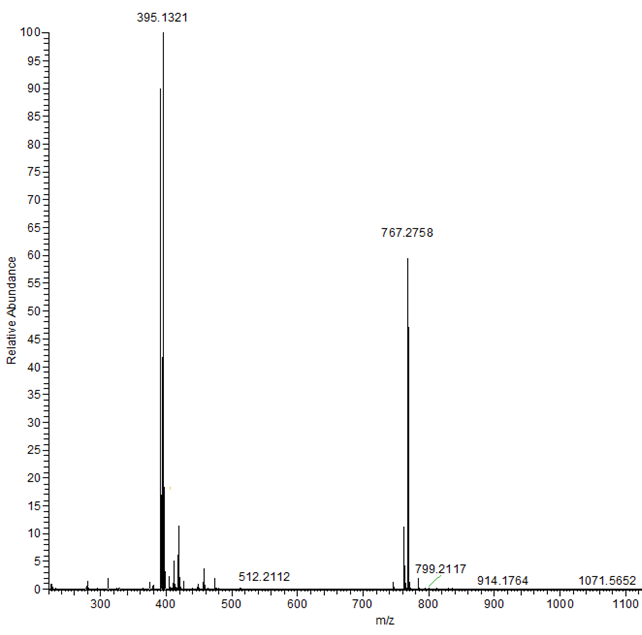


**Figure S1**: Positive-mode electrospray ionization mass spectrum (ESI-MS (+-mode)) of CL-1.

Figure S2: ^1^H NMR spectrum of CL-1.

*
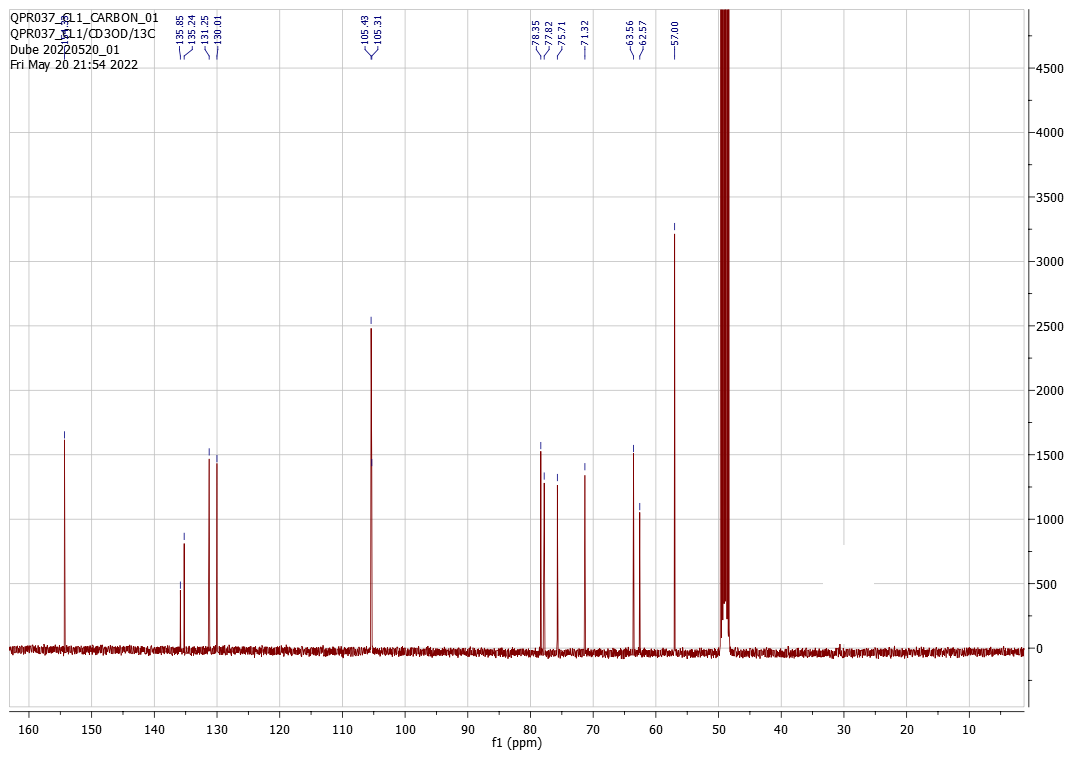
*

Figure S3: ^13^C-NMR spectrum of CL-1.

Figure S4: DEPT-135 spectrum of CL-1.
